# The “Floating Lotus” Sign on Intracardiac Echocardiography: Characterization and Predictors of a Novel Left Atrial Appendage Pseudo-Thrombus

**DOI:** 10.31083/RCM50367

**Published:** 2026-09-04

**Authors:** Liang Zhang, Yanyan Bai, Yanhua Zhu, Jianhua Sun, Liuliu Feng, Jingyu Zhang, Songqun Huang

**Affiliations:** ^1^Department of Cardiology, Third Affiliated Hospital of Naval Medical University, 200438 Shanghai, China; ^2^Department of Cardiology, Shidong Hospital, 200438 Shanghai, China; ^3^Department of Cardiology, Changhai Hospital, Naval Medical University, 200433 Shanghai, China

**Keywords:** intracardiac echocardiography, left atrial appendage, pseudo-thrombus, atrial fibrillation, catheter rotation maneuver

## Abstract

**Background:**

Intracardiac echocardiography (ICE) is increasingly used for left atrial appendage (LAA) assessment during atrial fibrillation (AF) procedures. We describe a novel, thrombus-mimicking artifact on ICE, termed the “Floating Lotus” sign.

**Methods:**

In this retrospective multicenter study, we analyzed data from 172 patients undergoing ICE-guided AF ablation and/or LAA occlusion. The incidence, imaging features, and clinical correlates of the “Floating Lotus” sign were assessed.

**Results:**

The “Floating Lotus” sign was observed in 48 patients (27.9%). It manifested as a mobile, echogenic mass within an anechoic space, revealing an underlying pericardial effusion. Patients exhibiting the sign had a higher prevalence of recent stroke (35.4% vs. 15.3%, *p* = 0.004), heart failure (64.6% vs. 8.1%, *p* < 0.001), and larger left atrial diameter (44.3 ± 6.8 vs. 39.7 ± 5.4 mm, *p* < 0.001). In multivariable analysis, only heart failure (left ventricular ejection fraction (LVEF) <55%) remained an independent predictor (odds ratio [OR] 14.39, 95% confidence interval [CI] 5.51–37.58, *p* < 0.001). Lower LAA emptying velocity was independently associated with persistent AF, higher CHA_2_DS_2_-VASc score, and recent stroke (all *p* < 0.05).

**Conclusion:**

The “Floating Lotus” sign is a common ICE pseudo-thrombus, often associated with heart failure. Accurate recognition of this sign, based on its dynamic imaging features, is therefore critical to prevent misdiagnosis and avoid unnecessary procedural cancellation or anticoagulation changes.

## 1. Introduction

Atrial fibrillation (AF), the most common sustained cardiac arrhythmia, significantly increases the risk of systemic thromboembolism. Notably, over 90% of thrombi in patients with non-valvular AF originate in the left atrial appendage (LAA) [1,2]. Therefore, meticulous exclusion of LAA thrombus is mandatory before any left atrial intervention, such as catheter ablation for AF or LAA occlusion, to prevent periprocedural stroke.

Transesophageal echocardiography (TEE) has long been established as the clinical gold standard for LAA thrombus detection [3]. However, TEE is a semi-invasive procedure associated with patient discomfort, requires conscious sedation, and carries a small but non-negligible risk of complications. Although cardiac computed tomography angiography (CTA) provides a non-invasive alternative, its utility is limited by a notably high false-positive rate for thrombus diagnosis, primarily due to inadequate LAA filling with contrast medium or low-flow states mimicking thrombus [4].

Intracardiac echocardiography (ICE) has emerged as a valuable real-time imaging modality for guiding AF ablation procedures. It provides high-resolution imaging of cardiac structures, including the LAA, without the need for general anesthesia or a separate TEE exam [5,6,7]. Consequently, ICE is increasingly being utilized for intraprocedural LAA assessment. Nevertheless, the interpretation of LAA images via ICE is not without pitfalls [8,9,10]. In our clinical practice, we have frequently observed a distinctive, mobile, echo-dense structure within the LAA cavity on ICE that resembles a thrombus but is not confirmed as such by other modalities or clinical outcome. We have termed this distinctive finding the “Floating Lotus” sign.

Systematic data on the prevalence and characteristics of this ICE-specific pseudo-thrombus are lacking. If unrecognized, it could lead to the erroneous cancellation of planned procedures or unnecessary anticoagulation escalation. This multicenter study aimed to characterize the incidence, imaging features, and clinical correlates of the “Floating Lotus” sign. We hypothesized that this sign represents a reproducible artifact arising from the visualization of the LAA wall floating within a pericardial effusion, specific to certain ICE imaging planes.

## 2. Method

### 2.1 Study Design and Population

This retrospective, multicenter study was conducted across three tertiary medical centers and included 172 consecutive patients with AF who underwent ICE-guided catheter ablation and/or left atrial appendage occlusion (LAAO) between January and December 2024. Patients were included if they had a confirmed diagnosis of non-valvular AF (paroxysmal or persistent) and underwent complete ICE imaging during the procedure. Patients were excluded if they met any of the following criteria: (1) confirmed intracardiac thrombus on TEE or left atrial CTA; (2) significant valvular heart disease (moderate or severe stenosis/regurgitation) or a history of cardiac surgery; (3) incomplete clinical or imaging data. The study protocol was approved by the institutional review boards of each participating center, and the requirement for written informed consent was waived due to the retrospective nature of the study, in accordance with local regulatory guidelines.

### 2.2 Data Collection

Demographic, clinical, and echocardiographic parameters were extracted from electronic medical records. Collected variables included age, sex, AF subtype (paroxysmal vs. persistent), CHA_2_DS_2_-VASc score (a validated clinical predictor of thromboembolic risk), left ventricular ejection fraction (LVEF), left atrial diameter (LAD), and history of ischemic stroke within the previous 3 months. The presence of LAA pseudo-thrombus and LAA flow velocity was assessed using ICE, as described below.

### 2.3 ICE Imaging Protocol and Definitions

All procedures were performed under ICE guidance using a 9-Fr ViewFlex™ Xtra ICE catheter (Abbott Medical, St. Paul, MN, USA) which was inserted via the femoral vein. Following vascular access, the catheter was positioned in the right atrium, and a clockwise rotation from the standard “Homeview” was performed to obtain optimal visualization of the LAA. When necessary, catheter shaping with a posterior or rightward tilt was employed to enhance visualization of the LAA orifice. Pulsed-wave Doppler was utilized to measure the LAA peak emptying velocity, with the sample volume placed at the LAA orifice.

To achieve the specific view for LAA assessment, the catheter was then advanced into the right ventricle. Under real-time guidance, it was rotated clockwise to visualize the pulmonary artery and gently advanced across the pulmonary valve. From this position, a counter‑clockwise rotation yielded a short‑axis view of the LAA, which frequently revealed a distinct echogenic, mobile, shaggy mass undulating within an anechoic space, simulating a thrombus. This specific imaging finding was termed the “Floating Lotus” sign (Fig. 1). This sonographic appearance corresponds to the distal LAA floating within the pericardial effusion. Key features distinguishing this LAA pseudo-thrombus from a true thrombus are as follows:

1. Absence of visible pectinate muscles along the margins of the anechoic space;

2. Continuous clockwise rotation of the ICE catheter demonstrates gradual merging of the “mass” into the LAA wall with clear visualization of underlying pectinate muscles, while a small pericardial effusion persists external to the LAA wall.

**Fig. 1.**
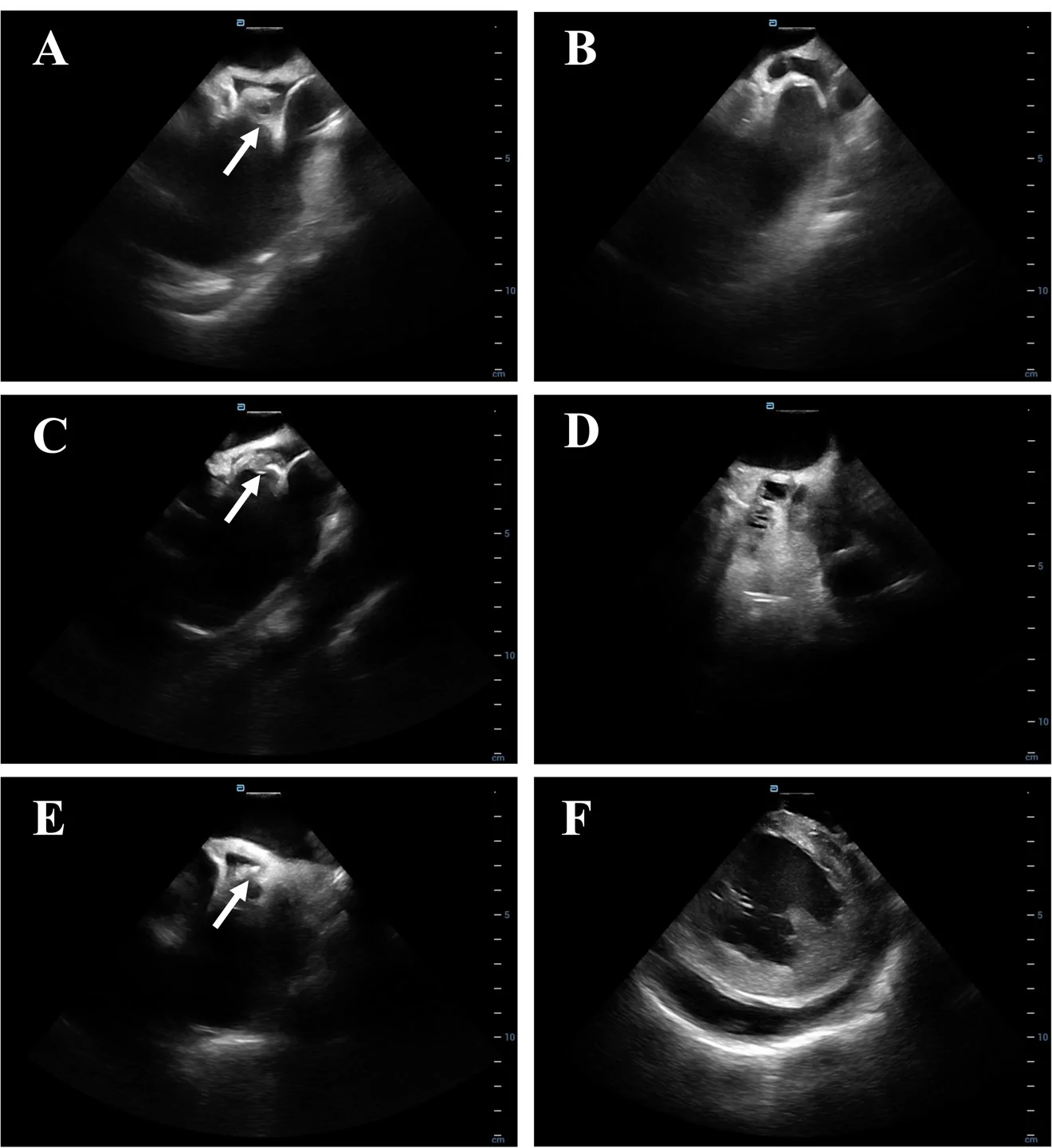
**The “Floating Lotus” sign observed on intracardiac echocardiography**. White arrows indicate the “Floating Lotus” structure. (A) An initial ICE view showing a distinct echogenic, mobile, shaggy mass undulating within an anechoic space, initially suggestive of a LAA thrombus. (B) Continuous clockwise rotation of the ICE catheter from the position in A demonstrates gradual merging of the mass into the LAA wall, revealing its pseudo-thrombotic nature. (C) Another example of the “Floating Lotus” sign in a different patient. (D) Further clockwise rotation of the ICE catheter from the view in C clearly visualizes the continuity of the structure with the LAA wall, along with underlying pectinate muscles, while a small pericardial effusion is seen external to the LAA wall. (E) A third instance of the “Floating Lotus” sign. (F) In the same patient as (E), a notable pericardial effusion is evident. This series illustrates the dynamic appearance of the LAA floating within pericardial fluid—a key feature distinguishing this pseudo-thrombus from true LAA thrombus. ICE, intracardiac echocardiography; LAA, left atrial appendage.

### 2.4 Statistical Analysis

Continuous variables are expressed as mean ± standard deviation if normally distributed. Categorical variables are presented as frequencies and percentages. Group comparisons were performed using the independent Student’s *t*-test for continuous variables, and the chi-square test for categorical variables, as appropriate. Potential predictors of the “Floating Lotus” sign were first assessed using univariable logistic regression, and significant variables (*p* < 0.10) were included in a multivariable logistic regression model. Univariable linear regression analysis was conducted to identify factors associated with LAA flow velocity. Variables with *p* < 0.10 in univariable analysis were included in a multivariable linear regression model using a stepwise (forward selection) method. All tests were two-tailed, and *p* < 0.05 was considered statistically significant. Statistical analyses were performed using SPSS version 26.0 (IBM Corp., Armonk, NY, USA).

## 3. Results

### 3.1 Study Population and Baseline Characteristics

A total of 172 consecutive patients with AF undergoing ICE-guided procedures constituted the study cohort, of which 72 were from Changhai Hospital, Naval Medical University, 55 from the Third Affiliated Hospital of Naval Medical University, and 45 from Shidong Hospital. The baseline clinical characteristics of the overall population, and stratified by the presence of the “Floating Lotus” sign, are summarized in Table 1. The cohort had a mean age of 65.7 ± 9.9 years, with 102 patients (59.3%) being male. Paroxysmal AF was diagnosed in 119 patients (69.2%), and persistent AF in 53 patients (30.8%). The mean CHA_2_DS_2_-VASc score was 2.6 ± 1.6. Heart failure, defined as LVEF <55%, was diagnosed in 41 patients (23.8%). The mean LAD was 41.0 ± 6.2 mm. A history of ischemic stroke within 3 months was present in 36 patients (20.9%). The “Floating Lotus” sign, indicative of LAA pseudo-thrombus, was observed in 48 patients (27.9%). The overall mean LAA peak emptying velocity was 54.0 ± 17.9 cm/s.

**Table 1.** **Baseline characteristics of the study population**.

Characteristic	Overall (N = 172)	“Floating Lotus” sign (+) (n = 48)	“Floating Lotus” sign (–) (n = 124)	*p *value
Age (years)	65.7 ± 9.9	65.4 ± 9.5	65.8 ± 10.2	0.798
Male, n (%)	102 (59.3)	26 (54.2)	76 (61.3)	0.394
Persistent AF, n (%)	53 (30.8)	19 (39.6)	34 (27.4)	0.121
CHA_2_DS_2_-VASc	2.6 ± 1.6	3.1 ± 1.8	2.7 ± 1.5	0.002
Stroke within 3 months, n (%)	36 (20.9)	17 (35.4)	19 (15.3)	0.004
Heart failure, n (%)	41 (23.8)	31 (64.6)	10 (8.1)	<0.001
LAD (mm)	41.0 ± 6.2	44.3 ± 6.8	39.7 ± 5.4	<0.001
LAA peak emptying velocity (cm/s)	54.0 ± 17.9	46.8 ± 19.9	56.8 ± 16.4	0.032

AF, atrial fibrillation; LAD, left atrial diameter; LAA, left atrial appendage.

### 3.2 Predictors of the “Floating Lotus” Sign

Compared to patients without the sign, those with the “Floating Lotus” sign had a higher prevalence of recent stroke (35.4% vs. 15.3%; *p* = 0.004) and heart failure (64.6% vs. 8.1%; *p* < 0.001), as well as a larger left atrial diameter (44.3 ± 6.8 mm vs. 39.7 ± 5.4 mm; *p* < 0.001). LAA peak emptying velocity was significantly lower in patients with the sign (46.8 ± 19.9 cm/s vs. 56.8 ± 16.4 cm/s; *p* = 0.032). However, when these significant univariable predictors were entered into a multivariable logistic regression model (Table 2), only the presence of heart failure (defined as LVEF <55%) remained an independent predictor (odds ratio [OR] 14.39, 95% confidence interval [CI] 5.51–37.58, *p* < 0.001).

**Table 2.** **Multivariable logistic regression analysis for predictors of the “Floating Lotus” sign**.

Variable	*p *value	Adjusted OR (95% CI)
Heart failure	<0.001	14.39 (5.51 to 37.58)
LAD (mm)	0.307	0.96 (0.89 to 1.04)
LAA peak velocity (cm/s)	0.498	1.01 (0.98 to 1.04)
Stroke within 3 months	0.586	1.38 (0.43 to 4.41)

OR, odds ratio; CI, confidence interval; Variables with *p* < 0.10 in univariable analysis were included in the multivariable model.

### 3.3 Determinants of LAA Peak Emptying Velocity

Univariable linear regression revealed that persistent AF, higher CHA_2_DS_2_-VASc score and stroke within 3 months were all significantly associated with lower LAA peak emptying velocity (Table 3). In the subsequent multiple linear regression model, these factors were still independently associated with reduced LAA flow velocity.

**Table 3.** **Univariable and multivariable linear regression analysis for factors associated with LAA peak emptying velocity**.

Variable	Univariable analysis	*p *value	Multivariable analysis	*p *value
	β Coefficient (95% CI)		β Coefficient (95% CI)	
Persistent AF	–5.29 (–10.39 to –0.18)	0.043	–6.95 (–12.02 to –2.65)	0.004
CHA_2_DS_2_-VASc	–2.46 (–4.51 to –0.41)	0.019	–2.11 (–4.32 to –0.61)	0.028
Stroke within 3 months	–16.34 (–23.97 to –8.70)	<0.001	–17.02 (–24.60 to –9.88)	<0.001
Age (year)	0.09 (–0.15 to 0.32)	0.469	-	-
Male sex	0.55 (–4.11 to 5.21)	0.816	-	-
LAD (mm)	–0.34 (–0.78 to 0.10)	0.133	-	-
Heart failure	–0.13 (–8.11 to 5.85)	0.75	-	-
“Floating Lotus” sign	–1.74 (–7.74 to 4.26)	0.567	-	-

β, unstandardized beta coefficient; CI, confidence interval; other abbreviations as in Table 1. Variables with *p* < 0.10 in univariable analysis were included in the multivariable model.

## 4. Discussion

This multicenter, retrospective study is the first to systematically describe the “Floating Lotus” sign, a distinctive pseudo-thrombus observed on ICE during LAA assessment. The key findings are: (1) this sign was prevalent, observed in 27.9% of our cohort; (2) it is characterized by a dynamic, shaggy, echogenic mass that merges with the LAA wall upon catheter rotation, with an underlying pericardial effusion being a constant feature; (3) the presence of heart failure (LVEF <55%) was the sole independent clinical predictor of this sign; and (4) while LAA flow velocity was lower in patients with the sign, it was not an independent discriminator in the multivariable model. Together, these findings delineate a novel and commonly encountered ICE artifact with a clear clinical predictor.

The robust association between the “Floating Lotus” sign and heart failure is mechanistically sound. Patients with heart failure frequently have elevated left atrial pressure and are more prone to develop pericardial effusion, even if subclinical [11]. We propose that this sign arises when the ICE catheter is positioned to obtain a short-axis view of the LAA from the right ventricle/pulmonary artery region. In this specific plane, the distal LAA wall, which is adjacent to the pericardial space, may be visualized tangentially. When surrounded by pericardial fluid, this thin, undulating LAA tissue can produce a mobile, shaggy, and apparently intracavitary echogenic mass—simulating a thrombus. The critical differentiating feature is the demonstration of continuity between this “mass” and the LAA wall upon minimal catheter manipulation, revealing the underlying pectinate muscles and confirming its extra-appendageal, pericardial location. This phenomenon is therefore likely unique to the ICE imaging environment, attributable to its proximity to the LAA-pericardial interface and specific imaging angles not commonly used in TEE.

Our findings carry significant clinical implications. Misinterpretation of the “Floating Lotus” sign as a true LAA thrombus could lead to the inappropriate cancellation of AF ablation or LAA occlusion procedures, or to unnecessary escalation of anticoagulation. Thus, awareness of this pseudo-thrombus is essential for interventional electrophysiologists and echocardiographers utilizing ICE. Operators should be familiar with the maneuver to obtain the specific short-axis LAA view and, more importantly, should routinely perform gentle clockwise rotation of the ICE catheter when any suspicious mass is detected. The dynamic “merging” of the apparent mass with the LAA wall is a pathognomonic feature and should be actively sought to establish the diagnosis of this benign artifact. Conversely, a true thrombus would remain discrete and intracavitary despite changes in imaging plane.

Notably, our data indicate that standard Doppler assessment of LAA peak emptying velocity cannot reliably distinguish this pseudo-thrombus from a true thrombus. Although velocity was lower in the sign-positive group, a finding consistent with their higher heart failure burden, it was not an independent predictor after adjustment. This underscores that dynamic imaging characteristics, rather than Doppler flow velocity, are key to its differentiation from true thrombus. Our analysis of LAA velocity determinants reaffirmed established pathophysiology: lower velocities were independently associated with persistent AF, higher stroke risk (CHA_2_DS_2_-VASc score), and recent stroke [12,13,14]. This aligns with the well-understood paradigm that atrial dysfunction and blood stasis, hallmarks of persistent AF and heart failure, predispose individuals to thrombogenesis and subsequent embolic events.

## 5. Limitation

Our study has several limitations. First, its retrospective design may introduce selection bias. Second, the diagnosis of the “Floating Lotus” sign and the exclusion of true thrombus relied on ICE criteria and clinical follow-up, as concurrent TEE was not routinely performed. The absence of a procedural or follow-up thromboembolic event in sign-positive patients provides reassuring indirect validation, but prospective studies with systematic TEE comparison would strengthen the diagnostic accuracy. Third, the sample size, though adequate for initial characterization, may be insufficient to identify weaker predictors of the sign. Future larger studies are warranted to validate our findings and explore additional associations. Finally, the experience of the ICE operator can influence image acquisition and interpretation; however, the multicenter design mitigates this concern to some extent.

## 6. Conclusion

The “Floating Lotus” sign is a common and reproducible ICE artifact that mimics LAA thrombus. It is strongly associated with heart failure, likely mediated by concomitant pericardial effusion. Recognition of its characteristic dynamic behavior, specifically its merging with the LAA wall upon catheter rotation, is crucial to avoid misdiagnosis. Incorporating a targeted, rotational imaging maneuver to assess for this sign should be considered in the standard ICE evaluation of the LAA to enhance procedural safety and decision-making.

## Data Availability

The corresponding author can facilitate access to anonymized records for legitimate research endeavors upon justified request.
